# Severe Hypercalcemia as the Initial Presentation of a Neuroendocrine Carcinoma of Unknown Primary Site: A Case Report

**DOI:** 10.7759/cureus.6075

**Published:** 2019-11-05

**Authors:** Hafeez Shaka, Sairam Raghavan

**Affiliations:** 1 Internal Medicine, John H. Stroger Jr. Hospital of Cook County, Chicago, USA

**Keywords:** humoral hypercalcemia of malignancy, neuroendocrine carcinoma, carcinoma of unknown primary, paraneoplastic syndrome, parathyroid hormone related peptide

## Abstract

Hypercalcemia is a common paraneoplastic syndrome that is associated with many malignancies. Hypercalcemia develops in malignancies through various mechanisms. Parathyroid hormone-related protein (PTHrP) is secreted by malignancy involving the lungs, breast, colorectum, bladder, and, rarely, neuroendocrine tumors. This is a rare case of severe hypercalcemia as the initial presentation of a high-grade metastatic neuroendocrine tumor of an unknown primary site.

## Introduction

Paraneoplastic syndromes are becoming increasingly prevalent in medicine, as our understanding of these conditions grows. The endocrine system is most commonly affected by paraneoplastic syndromes [1]. Hypercalcemia is a common finding in patients with malignancies and is present in approximately 10% of patients with advanced cancer. It also represents a poor prognosis. There are several mechanisms by which this may happen, but the excess secretion of parathyroid hormone-related peptide (PTHrP) is thought to account for a majority of the cases [2]. We report a case of severe hypercalcemia caused by a high-grade neuroendocrine tumor of unknown primary.

## Case presentation

A 29-year-old African American lady was brought into the emergency department with abdominal pain and recurrent headaches for four weeks, dizziness for a week, and an episode of syncope. She had crampy abdominal pain that was mild and progressive, associated with constipation but no nausea, vomiting, or abdominal distension. Headaches were mainly frontal, lasting a few hours, and transiently relieved with Tylenol. She denied any visual changes, photosensitivity, or eye pain. She developed dizziness about a week prior, which was worsened on standing, and improved on lying down. She fell a few times. She had a syncopal event lasting a few minutes with altered mental status following awakening, hence she was brought for further evaluation. The review of systems was significant for a 10-pound weight loss in a month, palpitations, fatigue polydipsia, and polyuria.

On presentation, her vital signs were within normal limits, except for mild tachypnea. Physical examination showed an obese lady, who was alert and fully oriented, with dry oral mucosa, pale conjunctiva, and an enlarged neck mass.

Laboratory investigations showed moderate microcystic anemia, creatinine of 1.7 mg/dL (normal range 0.6-1.2 mg/dL), calcium of 21 mg/dL (normal range 8.5-10.5 mg/dL), phosphate of 2.2 mg/dL (normal range 2.5-4.5 mg/dL), and lipase of 450 U/L (normal range 5-55 U/L). Other investigations included parathyroid hormone (PTH) of 4.36 pg/mL (normal range 12-88 pg/L), 25 hydroxy vitamin D of <7 ng/mL (normal range 30-100 ng/mL), 1, 25 dihydroxy vitamin D of 18 ng/mL (normal range 18-72 ng/mL), PTHrP of 33 pg/mL (normal range 14-27 pg/mL), and TSH of 0.20 uIU/mL (normal range 0.34-5.60 uIU/mL)

**Figure 1:**
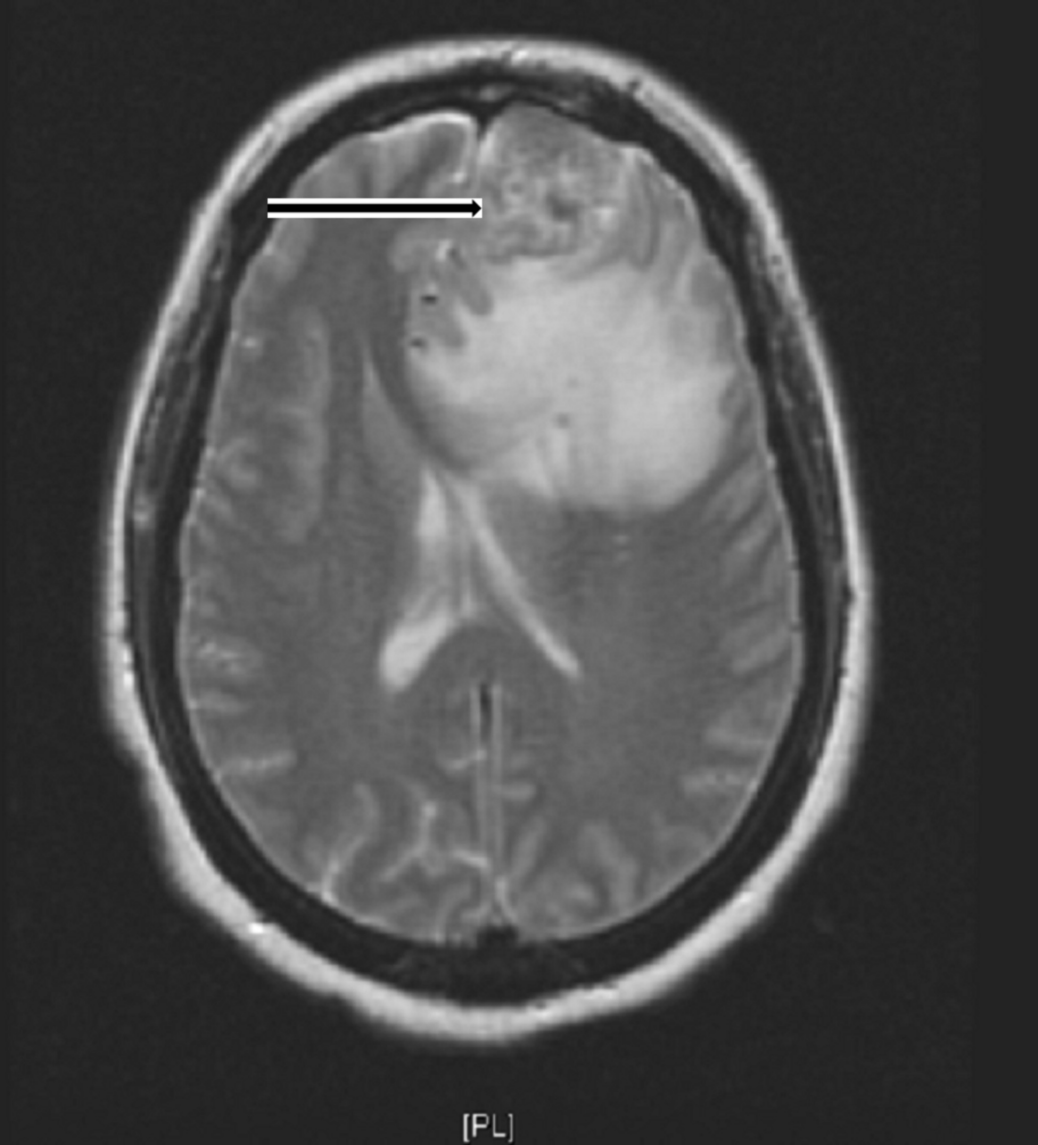
T2 Weighted MRI brain showing left frontal mass (black arrow) with surrounding vasogenic edema and midline shift MRI: magnetic resonance imaging

**Figure 2:**
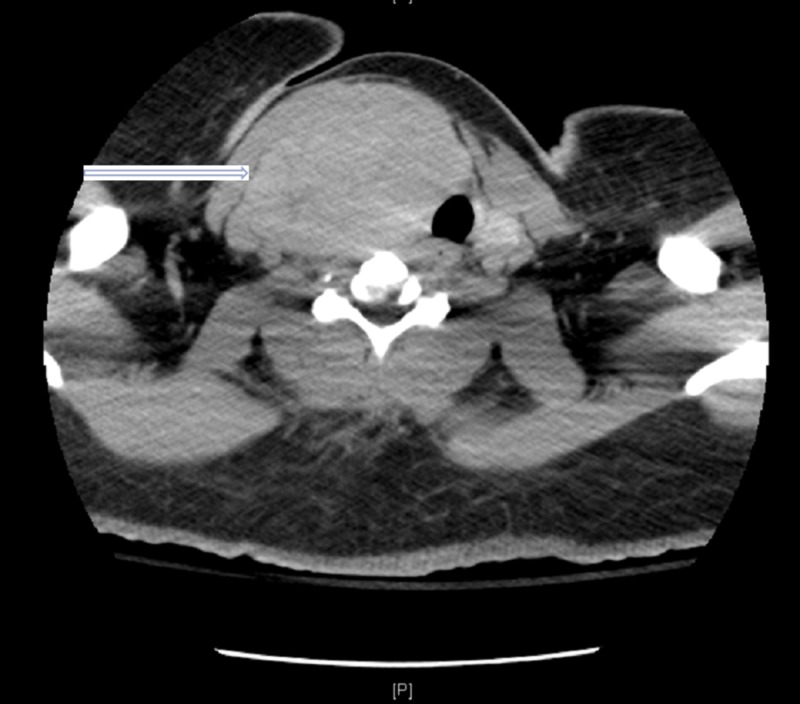
Axial view of CT neck with intravenous contrast showing right thyroid mass (white arrow) with tracheal deviation CT: computed tomography

**Figure 3:**
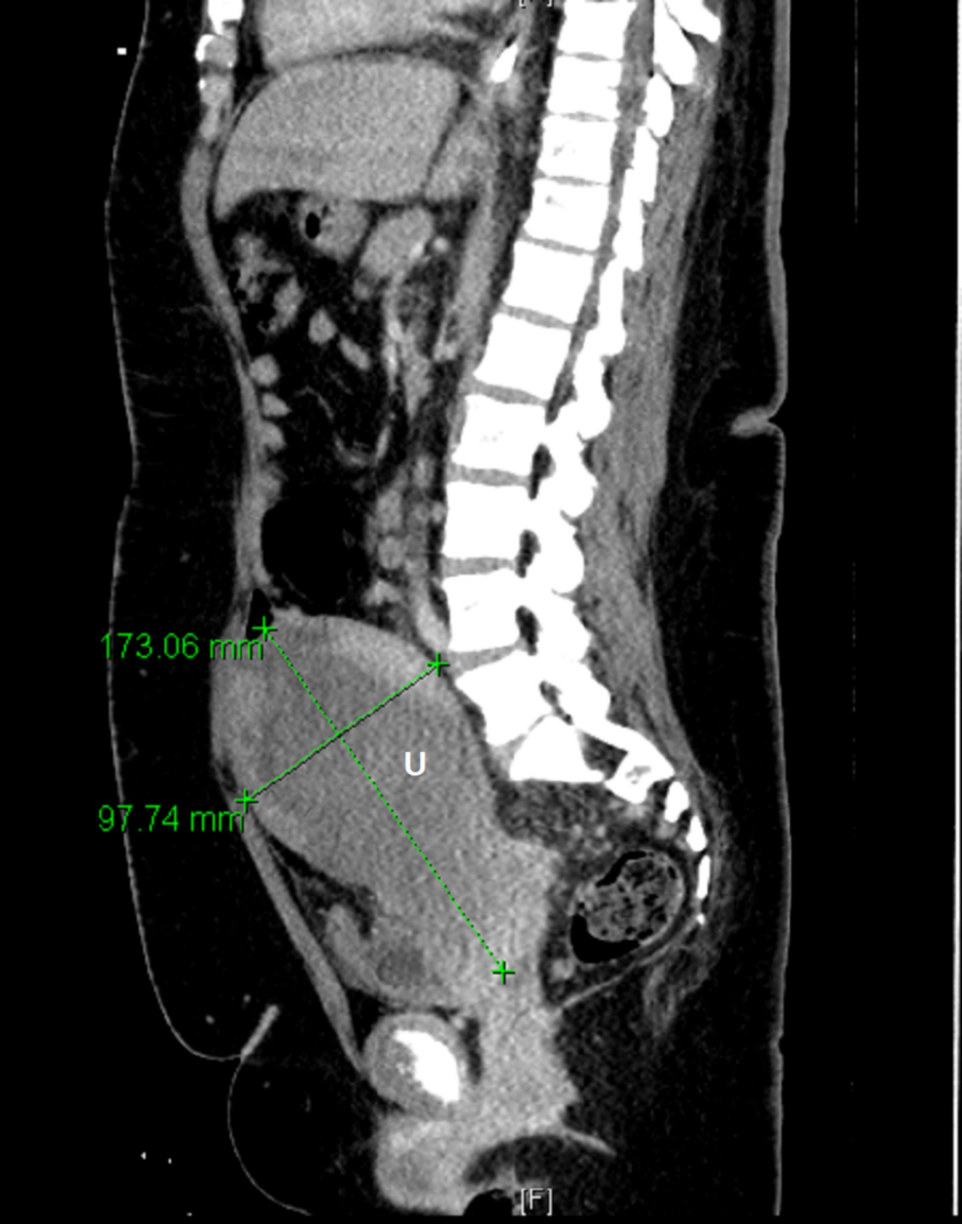
Sagittal view of CT abdomen and pelvis with intravenous contrast, showing large heterogeneous uterine mass (U) CT: computed tomography

Magnetic resonance imaging (MRI) of the brain showed a 3.6 cm dural-based left frontal lobe mass invading the inner table of the left frontal calvarium and extending into the left frontal sinus (Figure 1). A computed tomography (CT) scan of the neck showed marked heterogeneous enlargement of the right thyroid gland measuring approximately 8.7 x 5.2 x 11 cm (Figure 2). CT of the abdomen and pelvis showed an enlarged uterus with heterogeneous attenuation and enlarged endometrial canal, approximately 9.8 x 13.4 x 17.3 cm (Figure 3).

Further imaging revealed generalized lymphadenopathy, multiple hypodense liver lesions, infiltrations, and edema of the pancreatic head, and mixed lytic and sclerotic osseous lesions in the pelvis, with pathologic fracture of the right pubic ramus. An assessment of symptomatic severe hypercalcemia likely from metastatic neoplasm, acute interstitial pancreatitis, acute kidney injury, and brain lesion with mass effect was made and she was admitted into the intensive care unit for management. She received high-flow intravenous (IV) fluids, IV pamidronate, IV dexamethasone, and IV levetiracetam. Her calcium down-trended rapidly from 21 to 10.8 in three days of management, and she showed corresponding clinical improvement.

Multiple pathological results from biopsies of uterine, liver, thyroid, and brain masses showed high-grade neuroendocrine neoplasms of an unknown primary site.

## Discussion

Paraneoplastic syndromes can be the initial presentation of multiple malignancies. Hypercalcemia in malignancy is caused by four known mechanisms. The production of PTHrP is the most common mechanism, accounting for >80% of cases of hypercalcemia in malignancy and called humoral hypercalcemia of malignancy (HHM). Other mechanisms include osteolytic metastases causing excessive calcium release from the bone, the ectopic activity of 1-alpha hydroxylase enzyme, leading to excessive production of the active form of vitamin D, and ectopic production of PTH [3-5].

The majority of cases of HHM are associated with squamous cell carcinomas, renal, bladder, breast, ovarian, prostate, colorectal carcinomas, leukemia, and lymphomas [6]. Rarely, HHM has been identified in patients with neuroendocrine tumors of the pancreas [7-10], the esophagus [11], and the very rare ovarian non-small cell neuroendocrine carcinoma [12]. Our patient had HHM, diagnosed by severely elevated calcium, suppressed PTH, elevated PTHrP, and low vitamin D levels. Her clinical presentation was also likely due to hypercalcemia, with abdominal pain, polyuria, fatigue, and altered mental status in the setting of hypercalcemia being characteristic. These symptoms improved with the correction of hypercalcemia without definitive malignancy directed therapy.

The primary site of this biopsy-confirmed high-grade neuroendocrine tumor was difficult to establish. She had gross lesions in the brain, thyroid, liver, and uterus. Poorly differentiated high-grade neuroendocrine tumors can originate in the gastrointestinal tract, bladder, cervix, and prostate and due to rapid progression, management is warranted even when the primary site has not been established [13-14].

Although symptomatic HHM was the initial presentation leading to the diagnosis of the above metastatic neuroendocrine tumor, it is a marker of advanced disease and portends a poor prognosis [15-16].

## Conclusions

Severe hypercalcemia can be the initial presentation of high-grade neuroendocrine carcinoma, which is often mediated by PTHrP. This presentation is, however, a very poor prognostic marker.
